# Enhanced Recovery after Surgery in Kidney Transplantation: Shorter is Better

**DOI:** 10.3389/ti.2025.14899

**Published:** 2025-11-05

**Authors:** Thomas Poirier, Claire Garandeau, Gilles Blancho, Julien Branchereau

**Affiliations:** Centre Hospitalier Universitaire (CHU) de Nantes, Nantes, France

**Keywords:** kidney transplantation, ERAS in kidney transplantation, ERAS, kidney grafts, transplantation

Dear Editors,

Peri-operative management in kidney transplantation has not evolved in years, whereas in other fields it is constantly changing and improving. The implantation of Enhanced Recovery After Surgery (ERAS) in oncological urology have significantly improved postoperative outcomes by reducing complication rates, shortening hospital stays, accelerating functional recovery, and facilitating earlier initiation of adjuvant therapies, without compromising oncological safety, and has now become a standard in perioperative care for patients [1].

We aimed to transpose this approach to kidney transplantation, which is mostly an emergency procedure, except for living donor transplants. To this end, we conducted a historical-prospective study to assess the feasibility and effectiveness of an ERAS protocol in kidney transplantation at our center, which is a pioneer in the field of renal transplantation. There are few studies in the literature on this subjet [2–5].

We retrospectively analyzed a cohort of 130 patients who underwent their first kidney transplant without ERAS protocol. After analyzing this cohort, we implemented a multidisciplinary ERAS protocol in (Table 1) 2023 (surgical, nephrological, anesthetic, paramedical, with explanations for patients provided through documents and a patient video) and applied it to 130 consecutive patients hospitalized for their first kidney transplant. Living Donor (LD) transplant patients were also included (16% of the population, No-ERAS Group 15% vs. ERAS Group 16%, p = 0.86). For deceased donor, 73% were DNC and 27% DCC.

**TABLE 1:** Our eras protocol.

Pre-operative	Peri-operative	Post-operative
Oral and written information about kidney transplantation Oral, written and video information about ERAS protocol Regular physical activity Cessation of smoking and alcohol intake is recommended Risk stratification (Lee index, assessment of functional capacity, ASA score) Management of anemia Stabilization of chronical disease	Pre-surgery Pre-operative preparation of surgery site Carbohydrate loading till 6 h before surgery Oral fluids loading till 2 h before surgery Anesthesia Standard anesthesic protocol and depth of anesthesia monitoring (bispectral index, BIS) Multimodal analgesia including systematic TAP Block before or after surgery Neuromuscular blockade, monitoring and reversing Perioperative haemodynamic management (ballanced cristalloids, +/- minimally or non invasive cardiac output monitor *based on pulse contour analysis)* Preventing and treating postoperative nausea and vomiting Preventing intraoperative hypothermia (active warming device) Intraoperative glycaemic control No nasogastric intubation Anesthesic induction is performed via a peripheral venous cathter Central venous catheter is inserted if: - Induction with thymoglobulin >48 h and absence of arterio-veinous fistula - Poor venous capital, even if induction with thymoglobulin <48 h Surgery Systematic bladder catheterization before surgery Systematic ureteral stenting No systematic surgical drainage, and less drainage as possible	Early oral intake of fluids then solids after surgery according to protocol Early mobilization - POD 0: Edge of bed - POD 1: First step - POD 2: Walk and armchair - Mobilization exercise (according to protocol) Early recovery of normal bowel function - Chewing-gum - Limitation of opioid analgesia - Early mobilization - Early oral intake of fluids then solids after surgery according to protocol Own dress as soon as possible Multimodal opioid-sparing analgesia Early removal of intravenous infusion and treatment Early removal of surgical drainage - POD 2 if < 50 mL - Follow surgical instructions Early removal of bladder catheter - Women: POD 2 - Men: POD 4 - Follow surgical instructions Patient education about drugs (immunosuppression, analgesia …) Removal of ureteral stenting at 4–6 weeks in consultation Call of the patient at first day of discharge and nephrologic consultation at three-days then once a week

The two cohorts did not show any statistically significant differences, except for a higher rate of grafts on perfusion machines in the ERAS group (No-ERAS Group 54% vs. ERAS Group 68%, p = 0.027), which correspond to the increase in the use of perfusion machine for kidney graft in recent years. Median age was 57 years (44.8–67.2).

The implementation of this ERAS protocol led to a reduction in the median hospital stay (LOS) by 2 days (Non-ERAS 7 days vs. ERAS 5 days, p < 0.001). This reduction of LOS was also observed in both living donor (No-ERAS 6 days vs. ERAS 5 days, p < 0.001) and deceased donor subgroups (Non-ERAS 7 days vs. ERAS 5 days, p < 0.01). The reduction of LOS has been possible, without increasing postoperative morbidity excluding transfusion (Non-ERAS 12% vs. ERAS 16%, p = 0.37), transfusion rate (Non-ERAS 12% vs. ERAS 18%, p = 0.17), surgical re-intervention rate (Non-ERAS 10% vs. ERAS 8.5%, p = 0.67), or the rate of re-hospitalization before day 30 (Non-ERAS 15% vs. ERAS 24%, p = 0.086). There was, however, a trend toward increased re-hospitalizations among ERAS patients, with the majority (60%) being due to medical causes such as renal insufficiency (7 cases), infection (6 cases) or cardiac decompensation (4 cases). This may possibly be explained by the reduced length of hospital stay, as these complications now tend to occur at home, whereas they would have previously arisen during hospitalization. Graft outcomes, including time to recovery (median of 2 days), delayed graft function (14%), and graft failure rate (4.2%, 6.2% in the non-ERAS group vs. 2.3% in the ERAS group), were comparable between the ERAS and no-ERAS groups, with no statistically significant differences observed (p = 0.36, p = 0.82, and p = 0.12) respectively.

Thus, our ERAS protocol in kidney transplantation led to a reduction in hospital stay without increasing postoperative morbidity or early re-hospitalization rates. These positive results have allowed us to expand this protocol to all our kidney transplant patients, and will become our new standard in kidney transplantation care. There are still areas for improvement in kidney transplantation, as recently demonstrated by an American unit, with an awake kidney transplantation, with discharge on the next day.^1^ Although this is not yet published, this encouraging outcome may represent a potentiel future direction.

## Data Availability

The raw data supporting the conclusions of this article will be made available by the authors, without undue reservation.

## Footnotes

1
https://www.nm.org/healthbeat/medical-advances/new-therapies-and-drug-trials/Awake-Kidney-Transplantation-A-Revolution-in-Renal-Care
